# Microbial Diversity and Community Structure of Chinese Fresh Beef during Cold Storage and Their Correlations with Off-Flavors

**DOI:** 10.3390/foods13101482

**Published:** 2024-05-10

**Authors:** Zhiping Zhao, Ziqing Ling, Xin Nie, Dayu Liu, Hongfan Chen, Shengyuan Zhang

**Affiliations:** 1 Meat Processing Key Laboratory of Sichuan Province, College of Food and Biological Engineering, Chengdu University, Chengdu 610106, China; zhaozhiping@cdu.edu.cn (Z.Z.); lingziqing@stu.cdu.edu.cn (Z.L.); chenhongfan@stu.cdu.edu.cn (H.C.); zhangshengyuan@stu.cdu.edu.cn (S.Z.); 2 College of Food Science and Technology, Sichuan Tourism University, Chengdu 610100, China; niexin@sctu.edu.cn

**Keywords:** fresh beef, cold storage, microbial diversity, high-throughput sequencing, fungi, bacteria

## Abstract

To investigate the diversity and dynamics of microorganisms in Chinese fresh beef (CFB) without acid discharge treatment during cold storage, high-throughput sequencing was employed to analyze the CFB refrigerated for 0, 3, 7, and 10 days. The results showed that the community richness of the fungi and bacteria decreased significantly. However, the diversity decreased in the early stage and increased in the later stage. At the phylum level, Ascomycota (74.1–94.1%) and Firmicutes (77.3–96.8%) were the absolutely dominant fungal and bacterial phyla. The relative abundance of both fungal and bacterial phyla displayed a trend of increasing and then decreasing. At the genus level, *Candida* (29.3–52.5%) and *Lactococcus* (19.8–59.3%) were, respectively, the dominant fungal and bacterial genera. The relative abundance of *Candida* showed a trend of increasing and then decreasing, while *Lactococcus* possessed the opposite trend. KEGG metabolic pathways analysis suggested that carbohydrate metabolism, membrane transport, and amino acid metabolism were the major metabolic pathways of bacteria. Bugbase prediction indicated the major microbial phenotype of bacteria in CFB during cold storage was Gram-positive (17.2–31.6%). Correlation analysis suggested that *Lactococcus*, *Citrobacter*, *Proteus*, and *Rhodotorula* might be the main microorganisms promoting the production of off-flavor substances in CFB. This study provides a theoretical basis for the preservation of Chinese fresh beef.

## 1. Introduction

Beef is favored by consumers all over the world due to its high-quality source of protein and low-fat nutritional properties. In recent years, beef demand has increased globally [1]. China’s beef consumption has also significantly increased [2], and China’s beef production has grown from 6,440,600 tons to 7,182,600 tons, from 2018 to 2022. Currently, the consumption of meat including beef, pork, mutton, and poultry in China is mainly fresh meat, without acid discharge treatment at low temperature, which accounts for 60% [3]. Animals usually develop necrotic stiffness within three hours after slaughter, where cells respire anaerobically and thus accumulate lactic acid. Acid discharge treatment is a process in which the center temperature of the meat is lowered to 0–4 °C within 24 h after slaughtering, and the meat undergoes a sufficient cooling and maturation to slowly regain its softness. Fresh meat without acid discharge has better water retention and color properties, which is more in line with Chinese eating habits and cooking styles.

However, beef is susceptible to microbial contamination due to its rich nutrients, which can lead to spoilage and even produce toxins that harm consumers’ health [4]. Microbial spoilage of fresh meat is a common problem in the meat industry, and the total amount of meat rejected by consumers due to spoilage reaches millions of tons globally each year [5], leading to serious economic losses in the national economy.

Packaging methods and the slaughtering environment are important factors affecting the growth of spoilage microorganisms in fresh meat [6]. Hou et al. [7] investigated the preservation effect and microbial diversity of chilled fresh boneless beef knuckle packaged with different concentrations of carbon dioxide gas conditioning and found that *Pseudomona* and *Lactobacillus* were the dominant bacteria in the control group. Yang et al. [8] found that the microbial diversity of CO-modified atmosphere packaging (MAP) steaks was higher than that of 80% O_2_-MAP steaks, and *Brochothrix thermosphacta* and *Pseudomonas* spp. were the dominant bacteria in 80% O_2_-MAP steaks. Dorn-in et al. [9] identified the microorganisms that caused the deterioration of vacuum-packed refrigerated beef and found that lactic acid bacteria were the predominant bacteria including *Lactococcus piscium* and *Lactobacillus satsumensis*. On the other hand, the dominant fungi were *Cryptococcus curvatus*, *Candida famata*, *Trichosporon cutaneum*, and *Trichosporon montevidense*. Chen et al. [10] investigated the shelf-life and bacterial community dynamics of vacuum-packed beef stored under long-term super-chilled conditions from different slaughterhouses and demonstrated that there were significant differences in the initial flora composition and the bacterial succession in beef during storage from different slaughterhouses.

Chinese fresh beef (CFB) is still mainly fresh beef without acid discharge treatment at low temperature. Therefore, demonstrating the changes in the microbial genera in CFB during cold storage is of great significance for quality and safety control. However, there have been few reports on the microbial diversity of CFB during cold storage and the key microorganisms associated with the development of off-flavors in CFB. In this study, high-throughput sequencing (HTS) was performed to analyze the composition of the microbial flora in CFB without acid discharge during cold storage. Moreover, the correlation between the spoilage microorganisms and the off-flavors were investigated. This study provides a certain theoretical basis for the preservation of fresh beef and beef products.

## 2. Materials and Methods

### 2.1. Sample Preparation

Beef high rib was purchased from Ping’an Wholesale Market, Longquanyi District, Chengdu. The beef was cut into pieces of about 200 g per piece, sealed, and packaged in transparent high-barrier plastic bags (O_2_ permeability: 0.21 cm^3^/(m^2^ 24 h 0.1 MPa); water vapor permeability: 0.09 g/(m^2^ 24 h), with 4 groups placed on an ordinary plastic tray, and stored at 4 °C for 0, 3, 7, and 10 days, labeled as D0, D3, D7, and D10, respectively. For the determination of each sampling time, three bags of beef were mixed well, and three parallel experiments were performed for every mixed sample.

### 2.2. DNA Extraction and Sequencing

The total microbial genomic DNA was extracted according to the manufacturer’s instructions of the E.Z.N.A.^®^ soil DNA kit (Omega Bio-tek, Norcross, GA, USA), and the quality of the extracted genomic DNA was examined using 1% agarose gel electrophoresis. The quality of the extracted genomic DNA was determined using a NanoDrop2000 (Thermo Scientific Inc, Stoughton, MA, USA). The V3-V4 variable region of the bacterial 16S rRNA gene fragment and the fungal internal transcribed spacer region ITS gene fragment were amplified using upstream primers and downstream primers carrying barcode sequences [11]. For V3-V4 amplification, the primers were 338F (5′-ACTCCTACGGGGAGGCAGCAG-3′) and 806R (5′-GGACTACHVGGGGTWTCTAAT-3′). The fungal primers were ITS1F (5′-CTTGGTCATTTAGAGAGGAAGTAA-3′) and ITS2R (5′-GCTGCGTTCTTCATC GATGC-3′). Illumina MiSeq/NovaSeq high-throughput sequencing was performed by Shanghai Majorbio Bio-Pharm Technology Co., Ltd. (Shanghai, China).

### 2.3. Correlation between the Core Microbial Genera and Differential Off-Flavor Substances

Gas chromatography–ion mobility spectrometry (GC-IMS), equipped with a syringe and an autosampler unit for headspace analysis, was employed for the detection of off-flavor substances in a FlavourSpec^®^ from G.A.S. (Gesellschaft für Analytische Sensorsysteme mbH, Dortmund, Germany), as described in our previous study [12]. The ground beef samples were stored in aluminum foil bags at −40 °C for volatile flavor compounds analysis. Three grams of beef were placed in a 15 mL headspace flask. After 15 min incubation at a speed of 500 r/min at 60 °C, 500 μL of the headspace content was automatically injected by the heated syringe (85 °C). Chromatographic separation was performed on an MXT-WAX capillary column (30 m × 0.53 mm) at 60 °C with nitrogen (N_2_) as the carrier gas (purity ≥ 99.999%), at the following flow rate: 2 mL/min for 2 min, then a linear rise to 10 mL/min for 3 min, a rise to 15 mL/min for 10 min, to 50 mL/min for 5 min, and finally to 100 mL/min for 10 min. After separation in the capillary column, the headspace content was first injected into the ionization chamber for ionization, then through the shutter grid into the drift zone, and finally into the IMS detector. The experiment was carried out in triplicate.

The off-flavor substances were identified based on our previous study [12]. The correlation between the off-flavors and the core microbial genera was investigated using the Spearman correlation (*p* < 0.05, |R| > 0.6), performed by SPSS 27 (International Business Machines Corporation, Armonk, NY, USA). The correlation network diagrams were drawn using Cytoscape 3.9.1 (National Institutes of Health, Bethesda, MA, USA).

### 2.4. Data Processing

Based on taxonomic informatics, the changes in the microbial community structure during the spoilage process of CFB were investigated, and the data were visualized on the Meggie BioCloud platform (https://cloud.majorbio.com, accessed on 18 September 2023). The sequencing depth was analyzed using the Shannon index, and the microbial richness and diversity were assessed by α-diversity analysis. Venn diagrams were produced using the statistical software package R (3.3.1, R Foundation for Statistical Computing, Vienna, Austria). Circos figures were produced using the software Circos-0.67-7 (http://circos.ca/, accessed on 7 November 2023). Tax4Fun functional annotation was performed based on the Silva database, using the Tax4Fun (0.3.1) package of the R Programming Language (3.3.1, R Foundation for Statistical Computing, Austria) software. Bugbase phenotype prediction analysis was generated through the software BugBase (https://bugbase.cs.umn.edu/index.html, accessed on 7 November 2023). The raw data were summarized and processed using Excel 2021 (Microsoft Corporation, Redmond, WA, USA), and heatmaps were drawn using the R language (3.3.1).

## 3. Results and Discussion

### 3.1. α-Diversity Analysis

**Figure 1:**
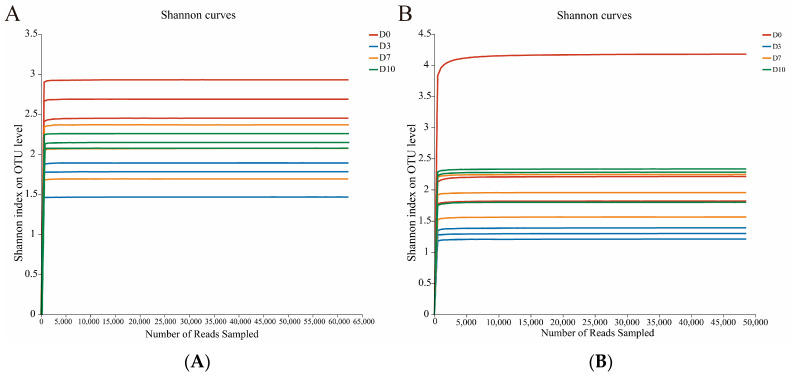
Fungal (**A**) and bacterial (**B**) Shannon index dilution curves in CFB with different refrigeration times.

The rarefaction curve deals with the sample coverage and depicts whether the sampling depth is sufficient or not to estimate the microbial diversity. The sample rarefaction curve of the Shannon’s index is shown in Figure 1. The fungi and bacteria had a total of 24 samples. With the extracted reads number increased, the curve showed a rapid increase and then a tendency to flatten out, indicating that the sequencing covered most of the fungi and bacteria in all the samples.

α-diversity can reflect the abundance and diversity of microbial communities, including a range of statistically analyzed indices to estimate species abundance and diversity. Species abundance is the number of individuals of each species in an area. Species diversity is a term used to define the different number of species in an area (species richness) and the abundance and distribution of these species in that ecosystem. The Sobs index is the actual observed value of the richness. The Shannon and Simpson indices were used to indicate the community diversity. The Chao1 and ACE indices were used to reflect the species richness, with higher scores indicating more species.

**Figure 2:**
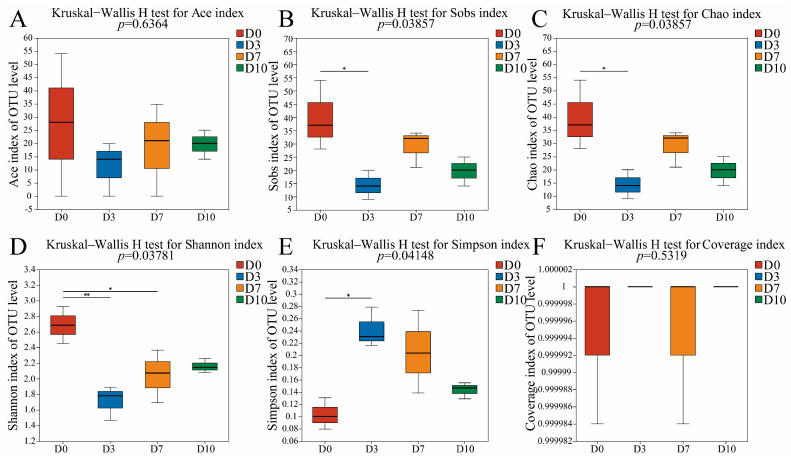
The α-diversity indices of fungi in CFB at different refrigeration times. * indicates significant difference (*p* < 0.05), ** indicates extremely significant difference (*p* < 0.01). (**A**) the Ace index, (**B**) the Sobs index, (**C**) the Chao index, (**D**) the Shannon index, (**E**) the Simpson index, (**F**) the Coverage index.

ACE, Chao1, and Sobs indices of fungi in D0 had the highest values (Figure 2), suggesting that the richness of fungi in D0 was significantly higher than that of other groups. Furthermore, the Simpson and Shannon indices indicated that the fungal diversity and homogeneity in D0 were significantly higher than that of other groups.

**Figure 3:**
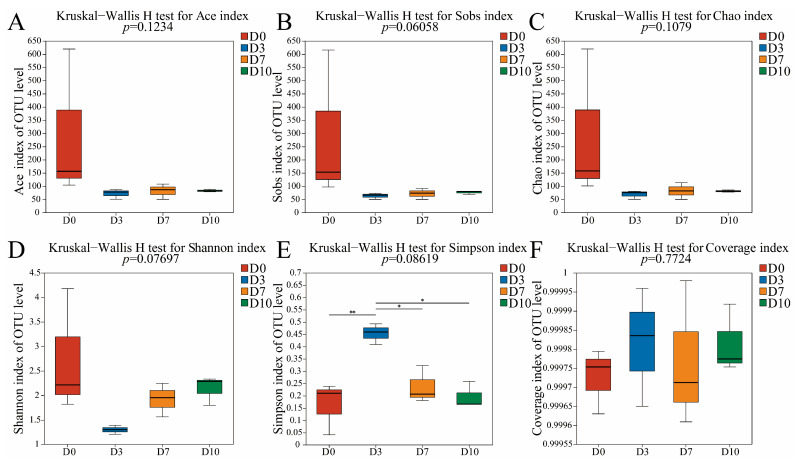
The α-diversity indices of bacteria in CFB at different refrigeration times. * indicates significant difference (*p* < 0.05), ** indicates extremely significant difference (*p* < 0.01). (**A**) the Ace index, (**B**) the Sobs index, (**C**) the Chao index, (**D**) the Shannon index, (**E**) the Simpson index, (**F**) the Coverage index.

Similarly, the bacterial ACE, Chao1, and Sobs indices were the highest in D0 (Figure 3), indicating that the highest species richness was found in CFB at 0 days of refrigeration. The species richness declined rapidly in the early period of refrigeration and then leveled off in the later period.

The microbial richness and diversity in D0 were markedly higher than those of the remaining three groups, which might be due to the microbial contaminations generated during a series of processes, such as slaughtering, transportation, sale, etc. [6,13]. Moreover, the growth of some of the microorganisms was suppressed during cold storage due to the stressful effect at low temperature [14,15] and the growth and reproduction of the psychrophilic dominant microorganisms. In this study, all samples had a good coverage (coverage > 0.999), indicating that the sequencing results could better reflect the microbial information in the samples.

### 3.2. OTU Venn Analysis

First, the 16S rRNA sequencing data analysis grouped the data into operational taxonomic units (OTUs) depending on the 97% identity threshold. OTUs can represent the microbial taxa; then, the analysis proceeded to the calculation and visualization of the diversity and composition of the microorganisms.

**Figure 4:**
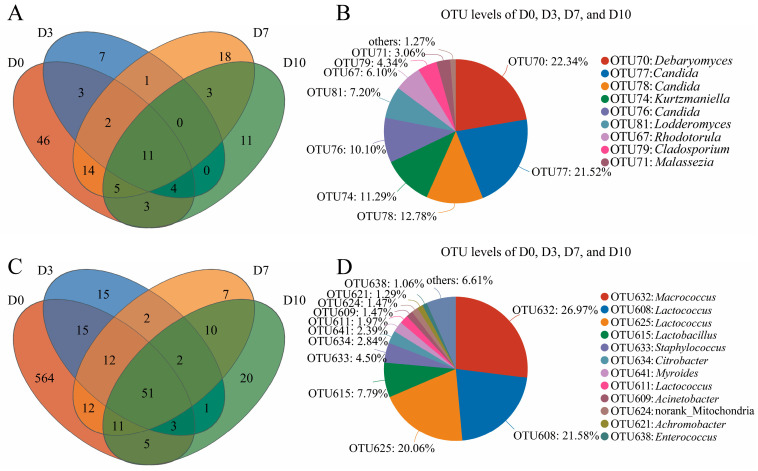
Venn diagram representation of shared and exclusive OTU sequences of fungi (**A**,**B**) and bacteria (**C**,**D**) in CFB samples.

The common core of the OTUs is represented by the overlapping areas in Venn diagrams, and each circle suggests the different samples. The Venn analysis of the fungal and bacterial OTU sequences in the CFB samples (D0, D3, D7 and D10) was performed, as depicted in Figure 4. Figure 4A shows the fungal OTU sequences, and a total of 128 OTU sequences were extracted from the CFB, of which 11 OTU sequences were core OTUs common to the four samples. The species with a relative abundance larger than 1% were *Candida* (44.40%), *Debaryomyces* (22.34%), *Kurtzmaniella* (11.29%), *Lodderomyces* (7.20%), *Rhodotorula* (6.10%), *Cladosporium* (4.34%), *Malassezia* (3.06%), and others, as shown in Figure 4B. Yeasts were the dominant spoilage fungi of CFB during cold storage, with *Candida* and *Debaryomyces* accounting for 44.4% and 22.34%, respectively. *Candida*, *Rhodotorula*, *Debaryomyces*, and *Trichosporon* are the common yeast genera found in fresh meat [16].

As seen in Figure 4C, in total, 730 bacterial OTU sequences were extracted from the D0, D3, D7, and D10 samples, of which 51 OTUs were shared core OTUs. From the 51 common OTUs (Figure 4D), a total of 10 core OTUs with an abundance greater than 1% were identified as follows: *Macrococcus* (26.97%), *Lactococcus* (43.61%), *Lactobacillus* (7.79%), *Staphylococcus* (4.50%), *Citrobacter* (2.84%), *Myroides* (2.39%), *Acinetobacter* (1.47%), g_norank_f_Mitochondria (1.47%), *Achromobacter* (1.29%), and *Enterococcus* (1.06%). *Lactococcus* and *Macrococcus* were the dominant bacterial genera causing CFB spoilage during cold storage. *Macrococcus* has a strong protein hydrolyzing activity and is able to degrade proteins and produce total volatile base nitrogen (TVB-N) resulting in the spoilage of beef [17,18]. It is well known that *Lactococcus* can grow at low temperatures and cause unpleasant flavors in refrigerated raw meat [19], which is a common dominant microbial genus in refrigerated beef [20].

### 3.3. Community Composition Analysis

**Figure 5:**
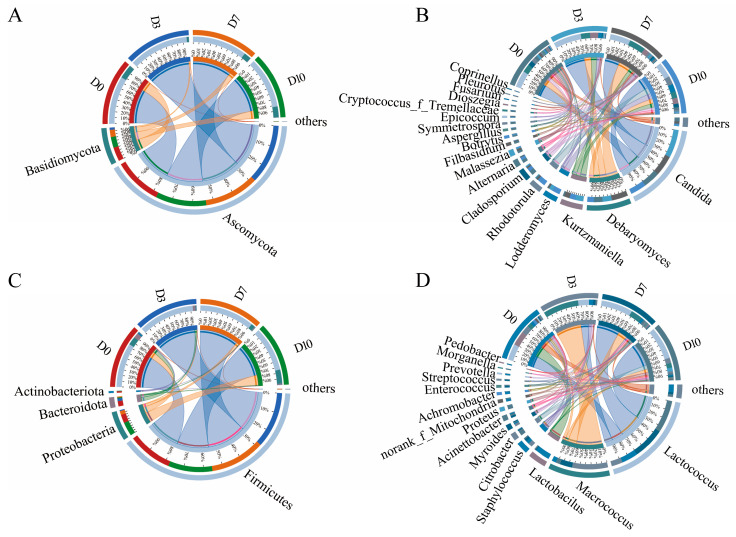
Circos figure of community structure in CFB at bacterial phylum (**A**) and genus level (**B**) and at the fungal phylum (**C**) and genus level (**D**).

The Circos plot reflects the composition proportion of each dominant species in the phylum and genus and visualizes the species trends across samples. The analysis of the fungal communities in the CFB at the phylum and genus levels during cold storage are shown in Figure 5. Ascomycota and Basidiomycota were the dominant phyla. Ascomycota richness accounted for more than 70% during cold storage, reaching 96% in the third day and then gradually decreasing to 82.6%. At the genus level, a total of 18 core fungal genera with a relative abundance greater than 1% were detected, namely *Candida* (29.3–52.5%), *Debaryomyces* (12.2–30.7%), *Kurtzmaniella* (2.2–15.4%), *Lodderomyces* (0.5–14.4%), *Rhodotorula* (0.6–15.4%), *Cladosporium* (1.9–8.6%), *Alternaria* (0–5.9%), *Malassezia* (1.3–4.4%), *Filobasidium* (0–4.3%), *Botrytis* (0–2.8%), *Aspergillus* (0.1–3.5%), *Symmetrospora* (0–3.5%), *Epicoccum* (0.6–1.1%), *Cryptococcus* (0–2.4%), *Dioszegia* (0–1.9%), *Fusarium* (0.1–1.1%), *Pleurotus* (0–1.1%), and *Coprinellus* (0–1.1%). Among them, *Candida*, *Debaryomyces*, and *Kurtzmaniella* were the dominant genera, and all their relative abundances showed an increasing and then decreasing trend. *Candida* is a cryophilic microorganism that can grow and reproduce at low temperatures [21], which was the absolutely dominant genus in CFB during cold storage. The lipase and protease produced by yeast have a significant role in promoting the discoloration as well as the production of off-flavors of meat products. In addition, some spoilage yeasts such as *Candida* and *Cryptococcus* are considered to be pathogenic yeasts, which can lead to human diseases [22].

The analyses of the bacteria at the phylum and genus levels are shown in Figure 5C and Figure 5D, respectively. Four bacterial phyla were identified at the phylum level, including Firmicutes, Proteobacteria, Bacteroidota, and Actinobacteriota. Among them, the relative abundance of Firmicutes accounted for more than 70% in all the CFB samples, which was the dominant phylum in the CFB during cold storage, consistent with the results of Hwang et al. [23]. A total of 15 core bacterial genera with an abundance of more than 1% were identified at the genus level, including *Lactococcus* (19.8–59.3%), *Macrococcus* (7.2–65.1%), *Lactobacillus* (0.4–26.3%), *Staphylococcus* (1.8–7.7%), *Citrobacter* (0.1–9.6%), *Myroides* (1.0–4.5%), *Acinetobacter* (0.3–3.6%), *Proteus* (0.01–6.7%), norank_f__Mitochondria (0.2–3.8%), *Achromobacter* (0.01–4.6%), *Enterococcus* (0.1–2.8%), *Streptococcus* (0.01–2.0%), *Prevotella* (0–2.6%), *Morganella* (0–1.4%), and *Pedobacter* (0–1.4%).

*Lactococcus* (29%) and *Lactobacillus* (26%) were the dominant bacterial genera in the CFB at the beginning of cold storage. With the extension of cold storage, *Macrococcus* showed an increasing and then decreasing trend, reaching the maximum abundance value of 65% in D3. *Macrococcus* is an aerobic microorganism; in the early stage of cold storage, its abundance increased by utilizing the oxygen in the barrier film package. However, with the depletion of oxygen, the growth of *Macrococcus* was inhibited, resulting in a decrease in the later stage. *Lactococcus* is a facultative anaerobe that grows well in an anaerobic environment. Therefore, when the oxygen in the package was completely consumed forming an anaerobic environment, *Lactococcus* gradually became the dominant spoilage organisms, which agreed well with the experimental results of Wen et al. [24] and Mansur [20] for vacuum-packed lamb and vacuum-packed beef, respectively.

### 3.4. 16S-Based KEGG Functional Pathway Prediction

The Kyoto Encyclopedia of Genes and Genomes (KEGG) is a database resource to understand high-level functions and utilities of the biological system. A total of 40 metabolic pathways were enriched by the 16S-based KEGG analysis. The abundance information of the top 15 metabolic pathways is shown in Table 1. The carbohydrate metabolism, amino acid metabolism, cofactors and vitamins metabolism, energy metabolism, signal transduction, lipid metabolism, xenobiotics biodegradation and metabolism, and terpenoids and polyketides metabolism in the CFB decreased during cold storage. However, membrane transport, nucleotide metabolism, translation, replication, and repair, and glycan biosynthesis and metabolism showed an overall increasing trend. Folding, sorting, and degradation, as well as the metabolism of other amino acids remained unchanged. Carbohydrate metabolism accounted for the largest proportion, more than 15% during cold storage. Bacteria utilize nutrients from beef for metabolic activities, and glucose is the preferred substrate utilized by most bacteria to convert nutrients into pyruvate through glycolysis [25], which is the precursor of spoilage molecules such as acetic acid, diacetyl, and acetylacetone [26,27]. When the glucose sugar is completely consumed, the bacteria will utilize the amino acids, nucleotides, and other nutrients in beef for metabolism. Microorganisms are able to utilize amino acids for metabolism to produce amines such as putrescine and cadaverine [28], leading to spoilage. Carbon dioxide generated from the metabolism of amino acids also contributes to the growth and multiplication of facultative anaerobic bacteria, which was consistent with the relative abundance of *Lactococcus* stored for 7 and 10 days. Membrane transport consists of three metabolic pathways, the ATP-binding cassette transporter (ABC transporter), the bacterial secretion system, and the phosphotransferase system, which could take up sugars by concentration gradient under the effect of adenosine triphosphate (ATP), the proton motive force, and the high-energy glycolysis intermediate phosphoenolpyruvate [29]. The ABC transporter is a type of transporting ATPase on the bacterial plasma membrane. The transporting ATPases constitute a large family of proteins possessing common functions and ATP-binding structural domains [30] and have a variety of transport functions including transporting nutrients into cells and toxic substances out of cells [31]. The bacterial secretion system performs transcytoplasmic membrane translocation in bacteria to deliver effector proteins to different classes of target cells and plays a key role in interbacterial competition and bacterial interactions with eukaryotic cells [32,33]. The phosphotransferase system is a widespread and efficient carbohydrate transport system in bacteria, which is the main pathway for carbohydrate uptake in bacteria [34]. The membrane transport reached a maximum relative abundance of 14.3% on the third day. Similarly, the relative abundance of *Macrococcus* also significantly increased on the third day, possibly due to an increase in the relative abundance of *Macrococcus* populations with secretion systems [35].

### 3.5. Bugbase Phenotype Prediction Analysis

**Figure 6:**
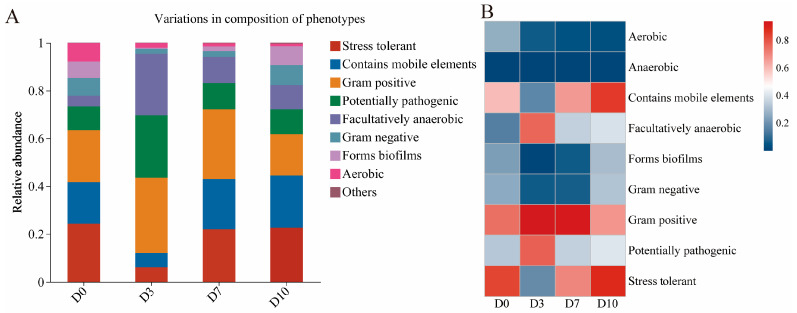
Bugbase phenotype classification statistics histogram (**A**) and heatmap (**B**) for bacteria in CFB.

BugBase is a microbiome analysis tool for estimating high-level phenotypes present in microbiome samples. The phenotypic prediction of bacteria present in the CFB was performed by using BugBase, as shown in Figure 6A. The predominant bacterial phenotypes in the CFB refrigerated for 0 days were stress tolerant, Gram-positive, and contained mobile elements, while Gram-positive, potentially pathogenic, and facultatively anaerobic phenotypes were predominantly present in the CFB refrigerated for 3 d. However, phenotypes that were Gram-positive, stress tolerant, and containing mobile elements were present in the CFB refrigerated for 7 and 10 d.

Gram-positive accounted for the highest proportion of the genera in refrigerated CFB, suggesting that Gram-positive bacteria were the main spoilage organisms during CFB refrigeration. In order to visualize the changes in the phenotypes in CFB during cold storage, heat map analysis of the phenotypes was performed, as revealed in Figure 6B. The Gram-positive phenotype was in higher abundance in the CFB during the cold storage period. The dominant genera *Lactococcus* and *Macrococcus* were both Gram-positive bacteria.

### 3.6. Correlation between the Core Microbial Genera and Differential Off-Flavor Substances

**Figure 7:**
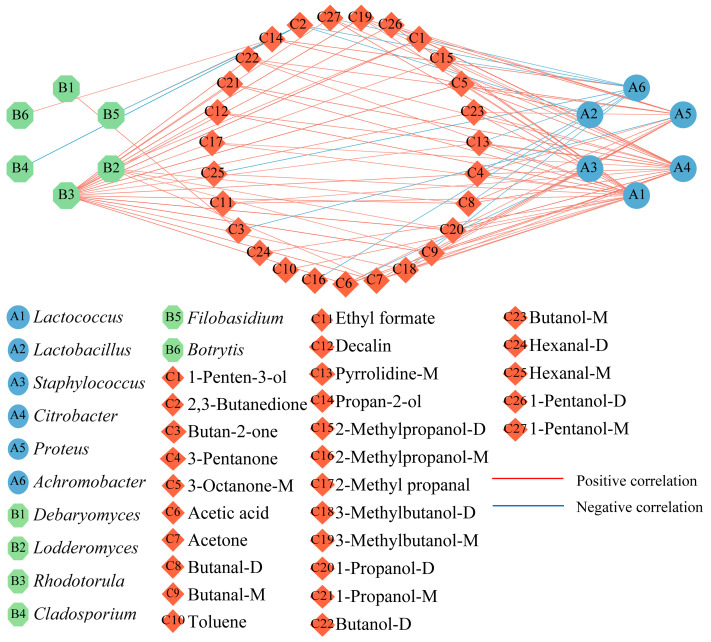
Correlations between microbial genera and off-flavor substances in CFB.

A total of 67 volatile flavor substances in CFB during cold storage were detected in our previous study [12], and 27 off-flavors were identified according to the aroma descriptions of the 67 substances in the present study [36], as shown in Table 2. The off-flavors could be used as potential biomarkers for judging the spoilage of CFB. The correlation between the top 10 microbial genera ranked in terms of the relative abundance of fungi and bacteria and the 27 off-flavors was investigated using the Spearman correlation (*p* < 0.05, |R| > 0.6), as shown in Figure 7. Six bacterial genera and six fungal genera were screened for a significant correlation with differential volatile flavor substances.

*Lactococcus* (A1) had a significant and positive correlation with 21 off-flavor substances such as decalin (R = 0.829, *p* < 0.01), 3-methylbutan-1-ol-D (R = 0.804, *p* < 0.01), and 1-pentanol-D (R = 0.821, *p* < 0.01), and some of the *Lactococcus* can produce butter and sour flavors in beef [19]. *Citrobacter* (A4) was significantly and positively correlated with 20 off flavors including 1-penten-3-ol (R = 0.918, *p* < 0.01), butanal-M (R = 0.863, *p* < 0.01), and 1-propanol-M (R = 0.829, *p* < 0.01). *Proteus* (A5) significantly and positively correlated with 14 off-flavor substances including 3-octanone-M (R = 0.832, *p* < 0.01), 1-propanol-D (R = 0.812, *p* < 0.01), and butanal-M (R = 0.812, *p* < 0.01). Butanal has a significant irritating odor, causing discomfort [37]. *Citrobacter* and *Aspergillus* both belong to the Enterobacteriaceae usually detected in fresh beef [38], which can decompose and utilize amino acids to produce amines and hydrogen sulfide [39]. *Rhodotorula* (B3) showed a significant and positive correlation with 18 off-flavor substances including butanal-M (R = 0.906, *p* < 0.01), 1-penten-3-ol (R = 0.915, *p* < 0.01), and 1-propanol-D (R = 0.916, *p* < 0.01). Yeast can utilize substrates for amino acid metabolism to produce higher alcohols with unpleasant odors such as n-propanol [40], leading to food spoilage.

## 4. Conclusions

The abundance of fungi and bacteria decreased significantly, and the diversity showed a decreasing and then increasing trend. *Candida* and *Lactococcus* were the main microorganisms responsible for the spoilage of CFB. The relative abundance of *Candida* increased and then decreased, while *Lactococcus* conversely decreased and then increased. The KEGG functional and phenotypic prediction analyses demonstrated that carbohydrate metabolism, membrane transport, and amino acid metabolism were the main metabolic pathways of bacteria in CFB. Gram-positive bacteria were the key microorganisms causing CFB spoilage. *Lactococcus*, *Citrobacter*, *Proteus*, and *Rhodotorula* were significantly and positively correlated with most of the off-flavors in CFB; thus, these four genera may be mainly responsible for the generation of off-flavors in CFB.

## Figures and Tables

**Table 1:** KEGG function prediction table.

KEGG Pathway_level2	Relative Abundance/%			
	D0	D3	D7	D10
Carbohydrate metabolism	15.6	15.7	15.2	15.4
Membrane transport	13.7	14.3	13.8	13.9
Amino acid metabolism	10.0	9.4	10.1	9.8
Nucleotide metabolism	7.2	8.4	7.9	8.1
Translation	6.7	7.9	7.4	7.6
Replication and repair	6.1	6.7	6.3	6.5
Metabolism of cofactors and vitamins	5.8	5.2	5.6	5.5
Energy metabolism	5.1	4.3	4.7	4.5
Signal transduction	4.9	4.1	4.4	4.2
Lipid metabolism	3.9	3.6	3.7	3.7
Glycan biosynthesis and metabolism	3.3	4.1	3.9	4.0
Xenobiotics biodegradation and metabolism	3.0	2.5	2.8	2.7
Folding, sorting, and degradation	2.7	2.7	2.7	2.7
Metabolism of other amino acids	2.7	2.7	2.7	2.7
Metabolism of terpenoids and polyketides	2.2	2.0	2.2	2.1
Others	6.9	6.4	6.6	6.5

**Table 2:** Description of off-flavor substances and their odors in CFB.

Number	Compound	CAS	Odor Description
1	1-Penten-3-ol	616-25-1	Butter, pungent, green
2	2,3-Butanedione	431-03-8	Butter
3	Butan-2-one	78-93-3	Ether
4	3-Pentanone	96-22-0	Ether
5	3-Octanone-M	106-68-3	Herb, butter, mold
6	Acetic acid	64-19-7	Acid, pungent, sour
7	Acetone	67-64-1	Pungent
8	Butanal-D	123-72-8	Pungent, green
9	Butyraldehyde-M	123-72-8	Pungent, green
10	Toluene	108-88-3	Paint
11	Ethyl formate	109-94-4	Pungent
12	Decalin	91-17-8	Pungent
13	Pyrrolidine-M	123-75-1	Alkaline
14	Propan-2-ol	67-63-0	Alcohol
15	2-Methylpropanol-D	78-83-1	Bitter
16	2-Methylpropanol-M	78-83-1	Bitter
17	2-Methyl propanal	78-84-2	Pungent, green
18	3-Methylbutan-1-ol-D	123-51-3	Burnt
19	3-Methylbutanol-M	123-51-3	Burnt
20	1-Propanol-D	71-23-8	Alcohol, pungent
21	1-Propanol-M	71-23-8	Alcohol, pungent
22	Butanol-D	71-36-3	Medicine
23	Butanol-M	71-36-3	Medicine
24	Hexanal-D	66-25-1	Fat, oil, green
25	Hexanal-M	66-25-1	Fat, oil, green
26	1-Pentanol-D	71-41-0	Balsamic, pungent, green
27	1-Pentanol-M	71-41-0	Balsamic, pungent, green

## Data Availability

The original contributions presented in the study are included in the article, further inquiries can be directed to the corresponding author.
